# Investigating geohazard risk in mountainous areas for underground gas storage using InSAR and development of a protocol for hazard prevention

**DOI:** 10.1371/journal.pone.0318860

**Published:** 2025-02-13

**Authors:** Siluo Lei, Jiawen Chen, Tingjun Wen, Elton J. Chen, Jiangen Xu, Ji Gao, Luyi W. Shen

**Affiliations:** 1 XiangGuoSi Natural Gas Storage Facility Company, PetroChina, Chongqing, China; 2 School of Civil and Hydraulic Engineering, Huazhong University of Science and Technology, Wuhan, China; 3 National Center of Technology Innovation for Digital Construction, Huazhong University of Science and Technology, Wuhan, China; 4 School of Petroleum Engineering, Chongqing University of Science & Technology, Chongqing, China; 5 Faculty of Engineering, China University of Geosciences, Wuhan, China; Guizhou University, CHINA

## Abstract

XiangGuoSi reservoir is a depleted gas reservoir that has recently (in 2014) been converted to an underground gas storage facility. It stores gas in the reservoir during the summer season and produces gas in the winter season. In this work, we present a case report on using InSAR to monitor the mountainous area beneath where the XiangGuoSi gas reservoir is located, along with its supporting pipeline infrastructures. Data, containing 159 scenes, from C-band Synthetic Aperture Radar (SAR) aboard Sentinel-1 satellite is used here, the processing period covered a timespan of 5.6 years. Importantly, we find that the surface deformation is not correlated with the reservoir’s gas injection/extraction history. This indicates that the gas storage’s underground operation does not increase geohazard risk in the area. Further, this indicates the reservoir rock’s pore structure is rather stable even during the cycles of injection/extraction, suggesting a stable reservoir performance even into the far future. Nevertheless, the natural movement of the mountain still poses a landslide risk for the pipeline structure. Given our observed deformation is mostly monotonically downward (subsidence) and in many points, linear, we develop a protocol using 1. the local maximum deformation rate point’s proximity to the pipeline and 2. the rate and total deformation magnitude reported during the monitoring period. After all, this report shows the capability of InSAR as a tool for mapping geohazards for mountainous areas where critical infrastructures are in the vicinity.

## Introduction

### InSAR for landscape monitoring

In the field of infrastructure and landscape monitoring, the integration of advanced remote sensing technologies, such as Interferometric Synthetic Aperture Radar (InSAR), marks a notable advancement [1–4]. InSAR is increasingly recognized for its contribution to the monitoring and maintenance of critical infrastructure [5–8]. It employs radar signals from satellites to generate high-resolution images capable of detecting minor changes on the Earth’s surface [9, 10].

InSAR provides precise deformation measurements across large areas and operates effectively under various weather conditions [11], both day and night. Its application in infrastructure monitoring is attributed to its accuracy and the non-invasive, cost-efficient approach [12, 13]. Continuous surveillance capabilities offered by InSAR often surpass the resolution of conventional ground-based monitoring techniques [14]. The utility of InSAR lies in its high sensitivity to subtle ground movements [15], which are often used as indicators for potential structural issues or instabilities and developing hazard prevention/mitigation protocols. By analyzing phase differences in radar signals reflected from the Earth’s surface over time [16], InSAR can achieve millimeter-level accuracy in detecting surface deformations [15]. This level of precision is critical for early warning systems, where the early detection of ground shifts can help prevent failures, thereby protecting public safety and mitigating economic losses. Moreover, InSAR’s capacity to monitor multiple structures simultaneously over large areas [17] offers significant advantages over traditional methods, particularly in urban environments or regions that are otherwise challenging to access. Regular maintenance checks, which can be logistically complex, become more manageable with this technology.

Integrating InSAR into infrastructure maintenance practices can extend the lifespan and enhance the safety of these structures while promoting a proactive approach to infrastructure management [13, 18]. Building upon the initial discussion of InSAR’s application in infrastructure monitoring, it is essential to examine its specific use in the surveillance of oil and gas reservoirs, as well as storage facilities. These sectors present unique challenges due to their dynamic nature and the critical importance of maintaining structural integrity for both environmental and safety considerations.

InSAR technology is particularly valuable in the oil and gas industry for monitoring subsurface movements associated with extraction activities [19–21]. The extraction of oil and gas often results in ground subsidence or uplift (often referred to as surface heave in the oil/gas sector), which can be precursors to more significant geological events. InSAR’s capability to detect and quantify these subtle ground deformations provides essential information for reservoir management. It assists in ensuring the structural stability of drilling platforms and surrounding infrastructure. Furthermore, InSAR data can inform the optimization of extraction processes by offering insights into reservoir behaviour, thereby improving efficiency and minimizing environmental impacts [22, 23].

Storage facilities, particularly those underground storage facilities for hazardous materials, require rigorous monitoring to prevent leaks and structural failures [24]. InSAR offers a non-intrusive method for continuous monitoring of these facilities. It can detect ground movements or changes in structural integrity that may indicate potential risks [25, 26]. This is particularly important for facilities such as underground natural gas storage areas, where surface deformations might signal issues such as leakage or pressure build-up.

InSAR is also critical for monitoring pipelines, especially those transporting oil and gas over long distances [27, 28]. These pipelines often traverse diverse terrains and are susceptible to ground movement, landslides, and other geohazards [29]. InSAR enables continuous surveillance of these extensive pipeline networks, detecting shifts or potential damages, thereby facilitating timely maintenance and preventing leaks and ruptures. InSAR data significantly contribute to disaster preparedness and emergency response strategies. By identifying areas of potential risk, such as unstable ground near storage facilities or along pipeline routes, InSAR allows operators to implement preventative measures and prepare more effective response plans in the event of an emergency [30].

In this paper, we discuss a case study involving InSAR monitoring of the XiangGuoSi underground natural gas storage facility, with particular attention to surface deformation and related geohazard impacts.

### Engineering and geological context

The XiangGuoSi gas field is situated in the mountainous region of Yubei District, Chongqing, where the topography is characterized by higher elevations in the north and lower elevations in the south [31]. The elevation in this area ranges from 400 to 820 meters, with the highest point reaching approximately 1,000 meters. The region is predominantly mountainous and sparsely populated, comprising agricultural land, tea gardens, and small coal mines. The area experiences frequent fog throughout the year and relatively low temperatures. Despite these conditions, transportation is facilitated by the Han-Yu Highway, rural roads, and internal roads within the gas field. Additionally, the Jinda gas transmission pipeline within the gas field provides an efficient route for gas extraction and transportation.

**Fig 1:**
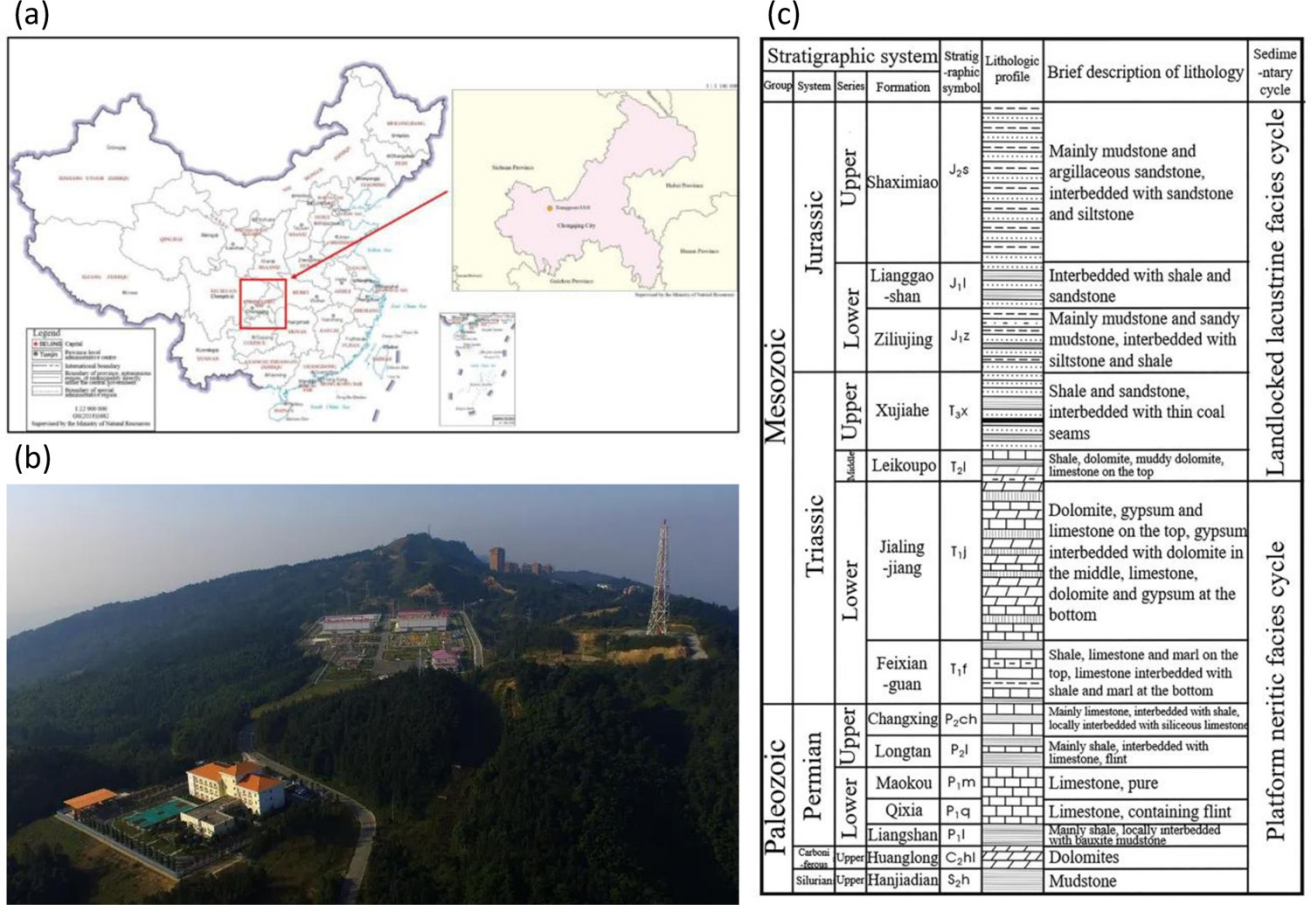
a) Geological locations of XiangGuoSi natural gas reservoir. b) An aerial view photo of the surface processing facility. c) the stratigraphical units near the XiangGuoSi reservoir area. a) and c) are adapted from [32].

The XiangGuoSi gas storage facility (Fig 1) is a converted structure from the original Carboniferous gas reservoir of the XiangguoSi gas field [32, 33]. The facility serves critical functions, including seasonal peak shaving and providing an emergency gas supply from Zhongwei to Guiyang and the Sichuan-Chongqing area. The storage facility project commenced in October 2011, with a successful trial injection conducted in June 2013. The initial construction phase included 12 injection and extraction wells, along with one gas extraction and monitoring well. An expansion project, completed in 2021, added 8 additional wells, including 7 injection and extraction wells and 1 gas extraction well.

Regarding regional tectonics, the XiangGuoSi structure is located within the Huaying Mountain structural group, in the central and steep structural zone of southeastern Sichuan. The structure is a narrow and asymmetric anticline, representing the easternmost broom-shaped branch of the Huaying Mountain anticlinal belt, commonly referred to as the Longwangdong Anticline [33]. It is bounded by the Yuelai Chang syncline to the west and the Tongluoxia Anticline to the east, separated by the Cizhu and Shaping synclines. To the north, it connects with the Sihai Mountain Anticline, forming a regular saddle shape, and dips into the Dadukou syncline in Chongqing to the south. The structure is characterized as a narrow "fault-block type" anticline controlled by an axial reverse fault, with the footwall of the east wing fault forming the Xiangdong concealed structure [32]. The overall structural trend follows a north-northeast direction. A more detailed depiction of the stratigraphical units can be found in Fig 1C.

The development of the Carboniferous gas reservoir in the XiangguoSi gas field can be categorized into five main stages [33]. The initial stage, from 1977 to 1980, marked the commencement of the reservoir’s development, beginning with the production of five gas wells, including Well Xiang 18, on November 14, 1977. By 1980, production reached 900,000 cubic meters per day, a rate maintained until 1987. Following this period, between 1987 and 1994, the gas field experienced a continuous decline in output, decreasing to 160,000 cubic meters per day by 1994. This reduction in production was likely due to a combination of complex geological and engineering factors. The subsequent period, from 1994 to 2004, was characterized by low pressure and low production, with a significant slowdown in the rate of decline. During this time, production decreased from 160,000 cubic meters per day to 55,000 cubic meters per day. The deceleration in the decline rate may be attributed to adjustments in development strategies or other factors.

After 2004, gas production stabilized at approximately 55,000 cubic meters per day, a stability that may be linked to the implementation of mature development strategies and technical optimizations. The closure stage occurred from 2010 to 2011, when the five production wells of the Carboniferous gas reservoir were sequentially closed due to the construction of the gas storage facility. Before closure, daily gas production was 56,000 cubic meters, with cumulative gas extraction totalling 4.024 billion cubic meters and total water production amounting to 1,903 cubic meters [34, 35].

As of April 25, 2019, following the initial successful injection test on June 29, 2013, eleven phases of "six injections and five extractions" have been completed [31, 33, 36]. The site is currently in its seventh injection period, with a cumulative total of 84.00×10^8^ cubic meters of gas injected and 54.04×10^8^ cubic meters of gas extracted.

## Data and methods

**Fig 2:**
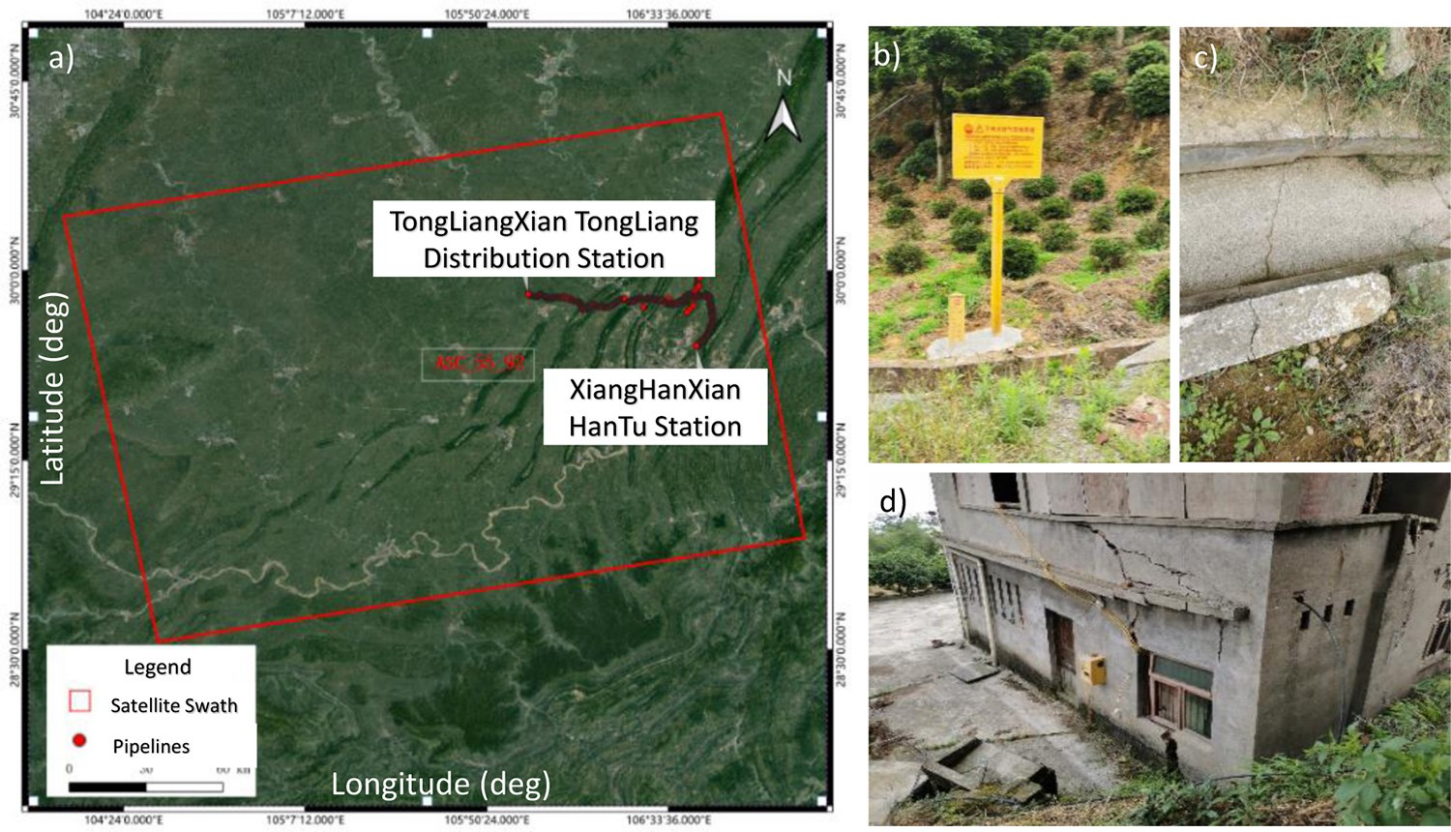
a) InSAR survey areas locations of the pipelines. b), c) and d) show the photos of local buildings with cracks caused by ground deformation.

The monitoring area for this project encompasses the geological hazard risks within, 1. The areas above the XiangGuoSi reservoir (Fig 2A) and 2. a 1-kilometre radius surrounding the gas pipelines supporting the underground gas storage. This area is known for having sporadical deformation and potential landslides. Some local houses, aqueducts etc., (Fig 2B and 2C), have observed cracks, due to ground movement and are deemed unfit for living. The primary focus is on 1. assessing the impacts of underground gas storage’s injection/extraction on the local geohazard risk; 2. assessing potential hazards such as landslides, collapses, mudslides, and unstable slopes along the pipeline route. The delineated monitoring area is illustrated in Fig 2 along with the encompassed pipelines. The site is managed by the XiangGuoSi Natural Gas Storage Facility Company and we are permitted to conduct our research onsite.

### Sentinel-1 satellite data usage in monitoring

The data used for this monitoring task are from the Sentinel-1 satellite. The Sentinel-1 satellite operates in a sun-synchronous orbit at an altitude of 693 km with an inclination of 98.18° [37]. It has an orbital period of 99 minutes and a revisit cycle of 12 days [38]. Towards the end of its lifespan, the satellite has an onboard data storage capacity of 900 Gbits. The telemetry and telecommand links utilize the S-band, with an uplink data transmission rate of 4 kbit/s and downlink data transmission rates of 16 kbit/s, 128 kbit/s, and 512 kbit/s. Data transmission is carried out using the X-band, with a rate of 600 Mbit/s [39]. Additionally, the Sentinel-1 satellite is equipped with a Laser Communication Terminal (LCT) for optical low Earth orbit to geostationary Earth orbit communication links. The LCT is based on the design of the TerraSAR-X (Land Synthetic Aperture Radar-X) satellite, with a power of 2.2 W, a telescope aperture of 135 mm, and uses the European Data Relay Satellite (EDRS) for downlink transmission of recorded data [39].

The C-band Synthetic Aperture Radar (SAR) aboard Sentinel-1 offers all-weather imaging capabilities and can provide high-resolution and medium-resolution data for land, coastal, and ice measurements. The combination of this all-weather imaging capability with radar interferometry allows for the detection of millimetre-level or sub-millimeter-level ground movement. The central frequency of the C-band in this Synthetic Aperture Radar is 5.405 GHz with a bandwidth of 0–100 MHz. It has a peak power of 4.368 kW, a pulse duration of 5–100 μs, and a pulse repetition frequency of 1000–3000 Hz. The radar’s antenna, weighing 880 kg (approximately 40% of the satellite’s launch mass), measures 12.3m × 0.84m. There are four operational modes for the onboard Synthetic Aperture Radar: Strip Map Mode (SM), Interferometric Wide Swath Mode (IW), Extra Wide Swath Mode (EWS), and Wave Mode (WV).

**Fig 3:**
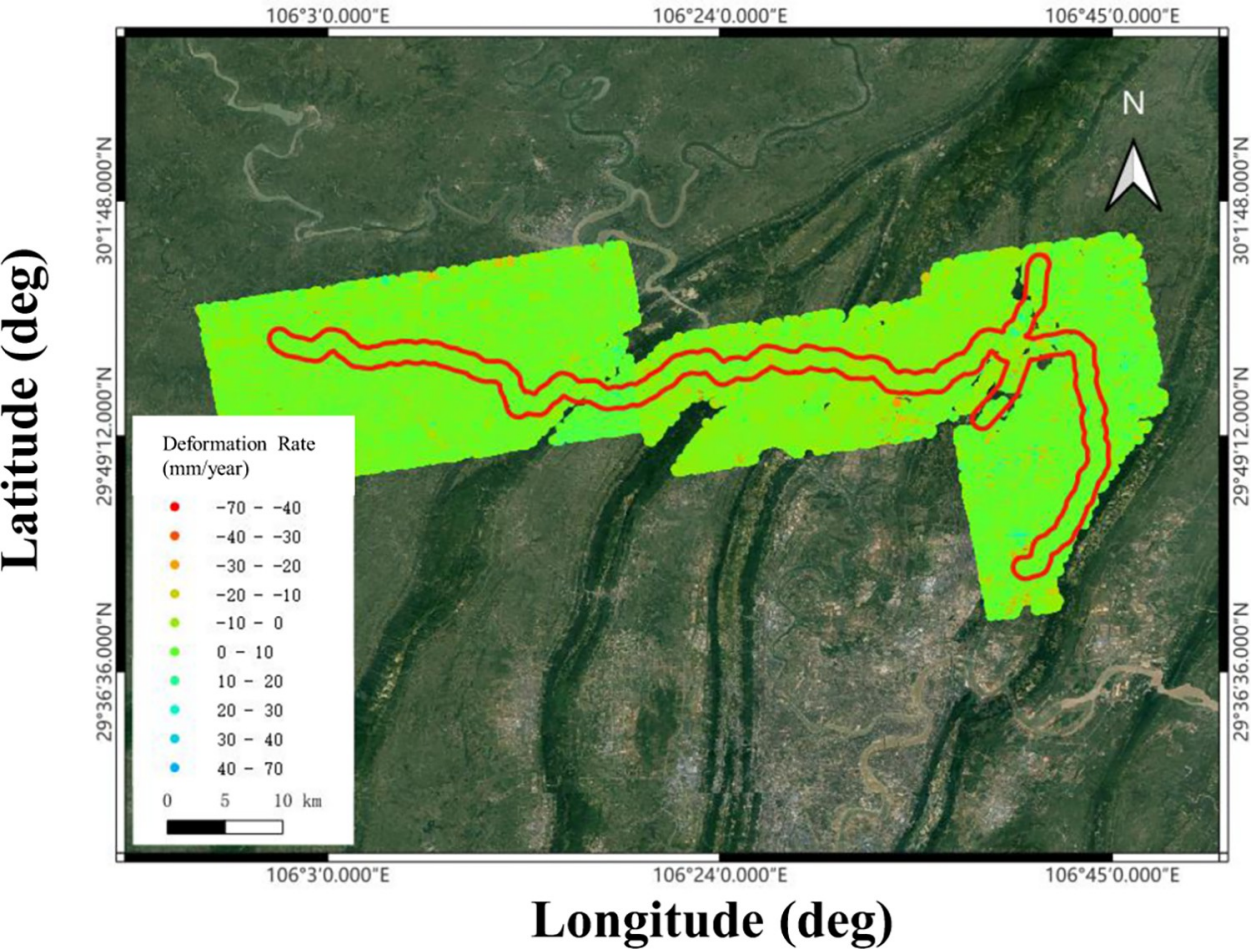
Ground deformation was revealed by InSAR for the areas near the XiangGuoSi underground storage with pipelines in the areas marked by the red solid lines.

The image acquisition period spanned from March 2017 to November 2022. These images were obtained from ascending orbits, totalling 157 scenes (Fig 3). Currently, the images are being collected at frequencies of every 12, 24, and 36 days. The coverage area of the ascending orbit is shown in Fig 3. A total of 157 images were processed. The specific dates of data collection are detailed in Table 1. The processing period covered a timespan of 5.6 years.

**Table 1:** Classification guidelines for risk levels.

Risk Level	Distance to the pipeline (m)	Classification guideline (deformation rate in mm/year and cumulative deformation in mm)	Suggested response
General attention	Greater than 25m	Rate ≤10	Action 1
	Less than 25m	Rate ≤20	
Continuous attention	Less than 25	10< Rate ≤20	Action 2
	Greater than 25	20< Rate ≤30	
Critical attention	Less than 25	Rate>20 or Cumulation>80	Action 3
	Greater than 25	Rate>30 or Cumulation>120	

### Data processing

**Fig 4:**
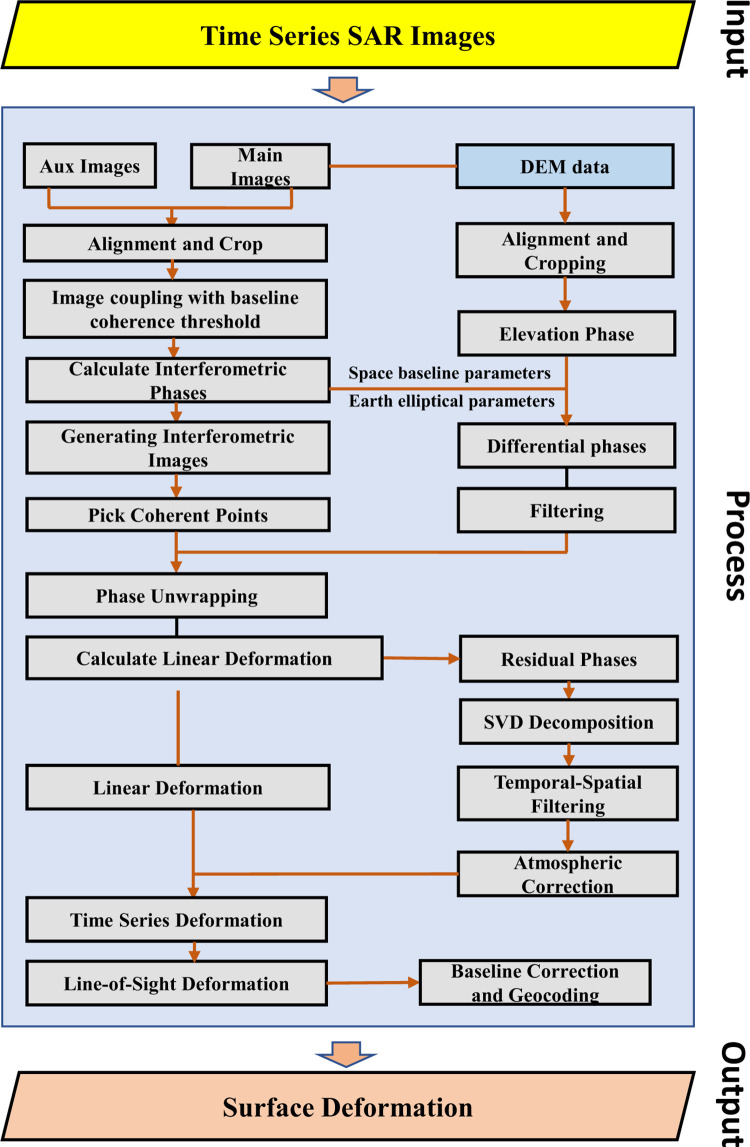
Workflow for the data processing techniques developed based on SBAS-InSAR.

This project utilizes a combined SBAS and PS-InSAR data processing approach to process the SAR data. This method enhances the accuracy of monitoring data and enables a deeper analysis of surface deformation risks and potential hazard sources in ten critical areas near the XiangGuoSi gas storage facility. At the core of InSAR data processing is the principle of radar interferometry [15], which is essentially the comparison of two or more SAR images of the same region, captured from slightly different viewpoints. Through this comparison, we calculate the phase difference between the images, which reflects the line-of-sight displacement of the ground between the acquisitions [40]. This process generates an interferogram—a complex image that encapsulates both the amplitude and phase difference of the radar signal. The phase information is particularly crucial, as it can be translated into precise measurements of surface deformation [41]. For a comprehensive overview of the data processing procedures, please refer to Fig 4.

The comprehensive process began with the preprocessing of 157 ascending orbit images (see S1 Appendix), which included converting SAR image data formats, extracting and geocoding monitoring areas, and registering the images to define the monitoring boundaries. To optimize processing, these images were divided into ten sections based on the boundaries of gas wells and pipeline ranges.

The Small Baseline Subset (SBAS) approach [40] for processing dense coherent scatterers is notable for its ability to establish interferometric pairs using multiple reference images from a set of input SAR scenes. This method offers a workflow for estimating both deformation rates and quantities. The process begins with the selection of multiple reference image interferometric pairs based on user-defined spatial and temporal baseline thresholds. Once initial interferometry, differencing, filtering, and unwrapping are completed, an external Digital Elevation Model (DEM) is employed for baseline refinement [42], followed by additional rounds of interferometric differencing and unwrapping.

Next, the procedure involves selecting a user-specified, gridded list of coherent points to conduct two-dimensional spatial searching and deformation estimation, ultimately determining optimal deformation rates and temporal deformation sequences. The SBAS approach for dense coherent scatterers addresses the limitations of traditional temporal interferometric SAR techniques (such as PSInSAR), improving both the spatial and temporal resolution of deformation estimation. Unlike PSInSAR, SBAS requires fewer SAR images (as few as seven scenes compared to PSInSAR’s 25) and places lower demands on spatial resolution.

Subsequent stages involved the processing of time-series permanent scatterer data (TSInSAR), essential for monitoring deformation points [43]. This phase included multilook processing, geocoding, selecting time-series points for permanent scatterers, and conducting differential interferometric processing [16]. Deformation was then calculated based on these points, followed by phase unwrapping and spatiotemporal filtering. The entire process also encompassed batch interferometry, differencing, filtering, and unwrapping of ten segmented datasets, along with dense coherent scatterer SBAS processing.

## Results

**Fig 5:**
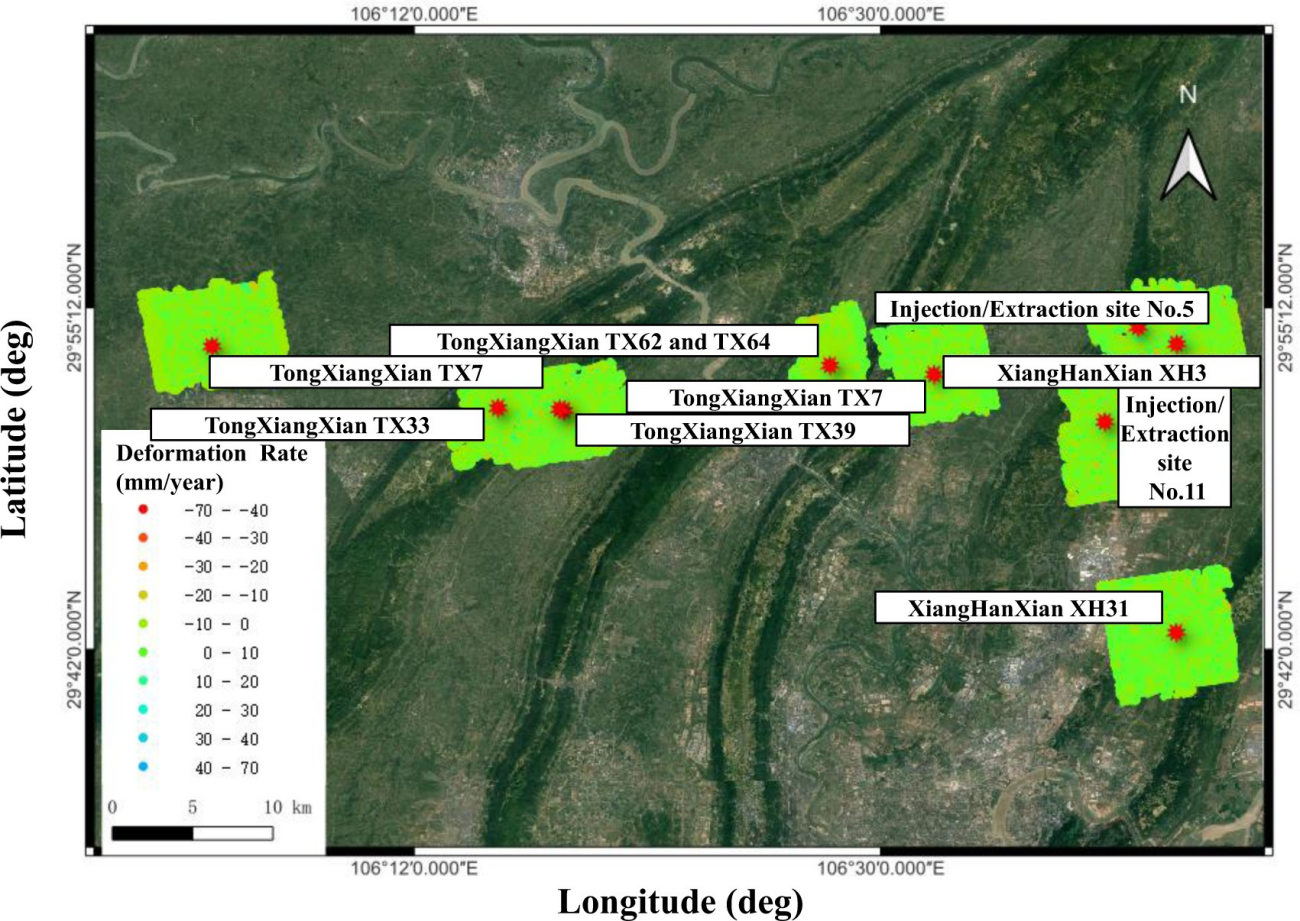
Locations of interest and the deformation measured for the areas near these points of interests.

The monitoring scope of this project encompasses potential geological disaster hazards on the areas of natural gas underground storage facility and within a 1-kilometer radius of 10 key areas surrounding the gas pipeline and process facilities managed by Xiangguosi Gas Storage Company. During the monitoring process, 10 geological hazard points were identified (Fig 5).

**Fig 6:**
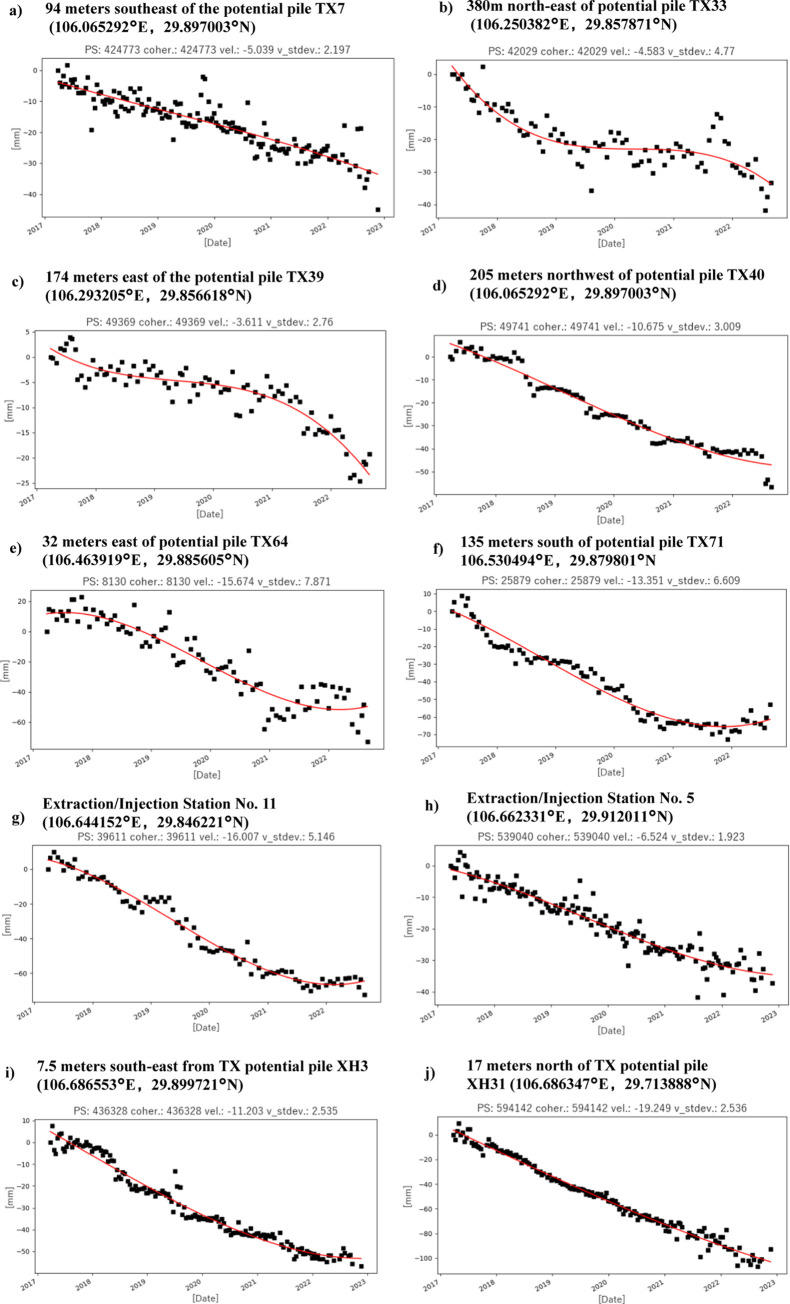
Temporal deformation series of the location of interests near the pipeline shown in Fig 5 and discussed in the main text.

Fig 6 shows the temporal deformation through the observation period, between March 2017 and November 2022, for these locations of interest. We fitted the results directly reported by InSAR with polynomial curves that can better represent the actual surface deformation of the areas. The gradients of the fitted polynomial curve also reveals the deformation rate that is used later as a critical criterion for determining the level of geohazard.

According to the monitoring results, deformation is observed in the area 94 meters southeast of the TongXiangXian potential pile TX7 (Fig 6A). The deformation rate in this area ranged from -13.08 mm/year to 4.35 mm/year. From the cumulative deformation profile around the hazard area, it is evident that the overall deformation in this area is not significant. The main subsidence areas are concentrated around points 4 and 5, with the maximum deformation point located at 106.065292°E, 29.897003°N, and the maximum cumulative subsidence amounting to -74.23 mm.

Regarding the TongXiangXian potential pile TX33, deformation has been detected 380 meters northeast of the pile (Fig 6B). The deformation rate in this area ranged from -9.33 mm/year to 1.04 mm/year. The overall deformation is minor, and the maximum deformation point is at 106.250382°E, 29.857871°N, with the maximum cumulative subsidence being -58.94 mm. This area, located in a depression within farmland, adjacent to the monitored pipeline, exhibits an intermittent subsidence trend. The subsidence trend was significant from March 2017 to June 2019, with an overall subsidence rate of -4.58 mm/year and a cumulative subsidence of -33.25 mm.

The area 174 meters east of the TongXiangXian potential pile TX39 shows deformation (Fig 6C), with a deformation rate ranging from -8.94 mm/year to 2.21 mm/year. Subsidence is widespread in this area, with the maximum deformation point at 106.293205°E, 29.856618°N and a maximum cumulative subsidence of -73.95 mm. Located on a hillside north of Qingyun Reservoir and adjacent to the monitored pipeline, this area shows a persistent subsidence trend. Subsidence was notable between January 2021 and July 2022, with an overall subsidence rate of -3.61 mm/year and a cumulative subsidence of -19.26 mm.

The area 205 meters northwest of TongXiangXian potential pile TX40 shows a deformation rate ranging from -11.26 mm/year to 1.35 mm/year (Fig 6D). The overall deformation is minor, and subsidence is primarily around point 6. The maximum deformation point is at 106.065292°E, 29.897003°N, with the maximum cumulative subsidence of -74.23 mm. Located on the top of a hill, approximately 24 meters from the monitored pipeline, this area exhibits a periodic subsidence trend, with significant subsidence each summer. The overall subsidence rate is -10.67 mm/year, and the cumulative subsidence is -56.70 mm.

Deformation in the area 32 meters east of TongXiangXian potential pile TX64 is notable (Fig 6E), ranging from -17.72 mm/year to 0.84 mm/year. The subsidence trend is clear and widespread, with the maximum deformation point at 106.464171°E, 29.885135°N, and a maximum cumulative subsidence of -91.91 mm. Located on a mountain slope above Baishui Creek and adjacent to the monitored pipeline, this area shows a persistent subsidence trend, with a subsidence rate of -15.67 mm/year and a cumulative subsidence of -72.96 mm.

The area 135 meters south of TongXiangXian potential pile TX71 shows a deformation rate ranging from -13.35 mm/year to 4.66 mm/year (Fig 6F). Subsidence trends are evident around points 3 and 4, close to the pipeline, with continued monitoring recommended. The maximum deformation point is at 106.530494°E, 29.879801°N, with a maximum cumulative subsidence of -52.99 mm. Located at the bottom of a hill slope and adjacent to the monitored pipeline, this area exhibits a phased subsidence trend. Significant subsidence occurred from November 2017 to January 2018 and from January 2020 to January 2021. The overall subsidence rate is -13.35 mm/year, and the cumulative subsidence is substantial.

The area around injection and extraction station No. 11 exhibits a deformation rate ranging from -19.33 mm/year to 7.13 mm/year (Fig 6G). Deformation is widespread with points 2 and 5 experiencing significant deformation. The maximum deformation point is at 106.621382°E, 29.856471°N, with a maximum cumulative subsidence of -106.74 mm. Located on a slope with fragmented land use patterns and adjacent to the monitored pipeline, this area exhibits a complex subsidence trend that has been accelerating.

According to the monitoring results, the deformation rate around Injection and Extraction Station No. 5 in the northern section ranged from -6.5 mm/year to 5.09 mm/year (Fig 6H). The cumulative deformation profile of the surrounding hazard area indicates that overall deformation in this region is relatively minor. The maximum deformation point is located at 106.662331°E, 29.912011°N, with the maximum cumulative subsidence reaching -37.16 mm. This area is situated on a mountain slope beside a road, approximately 54 meters from the monitored gas pipeline. The site exhibits a persistent subsidence trend with a rate of -6.52 mm/year and a cumulative subsidence of -37.16 mm.

The southeast area, 7.5 meters from potential pile XH3 on the XiangHan pipeline, experienced deformation rates between -11.76 mm/year and 6.21 mm/year(Fig 6I). The cumulative deformation profile highlights significant movement around points 5, 6, 7, and 8, with the maximum deformation point at 106.686553°E, 29.899721°N, and a maximum cumulative subsidence of -80.61 mm. Located on a mountain slope next to a road and adjacent to the monitored gas pipeline, this area shows a sustained subsidence trend with a rate of -11.2 mm/year and a cumulative subsidence of -56.74 mm.

Deformation is also evident in the area 17 meters north of potential pile XH31 on the XiangHan pipeline, with deformation rates ranging from -20.32 mm/year to 4.01 mm/year (Fig 6J). The cumulative deformation profile indicates a significant subsidence trend, with the maximum deformation point located at 106.686347°E, 29.713888°N. Situated on a slope between hills and closely adjacent to the monitored gas pipeline, this area exhibits a continuous subsidence pattern with a rate of -19.24 mm/year and a cumulative subsidence of -92.51 mm.

## Discussion

**Fig 7:**
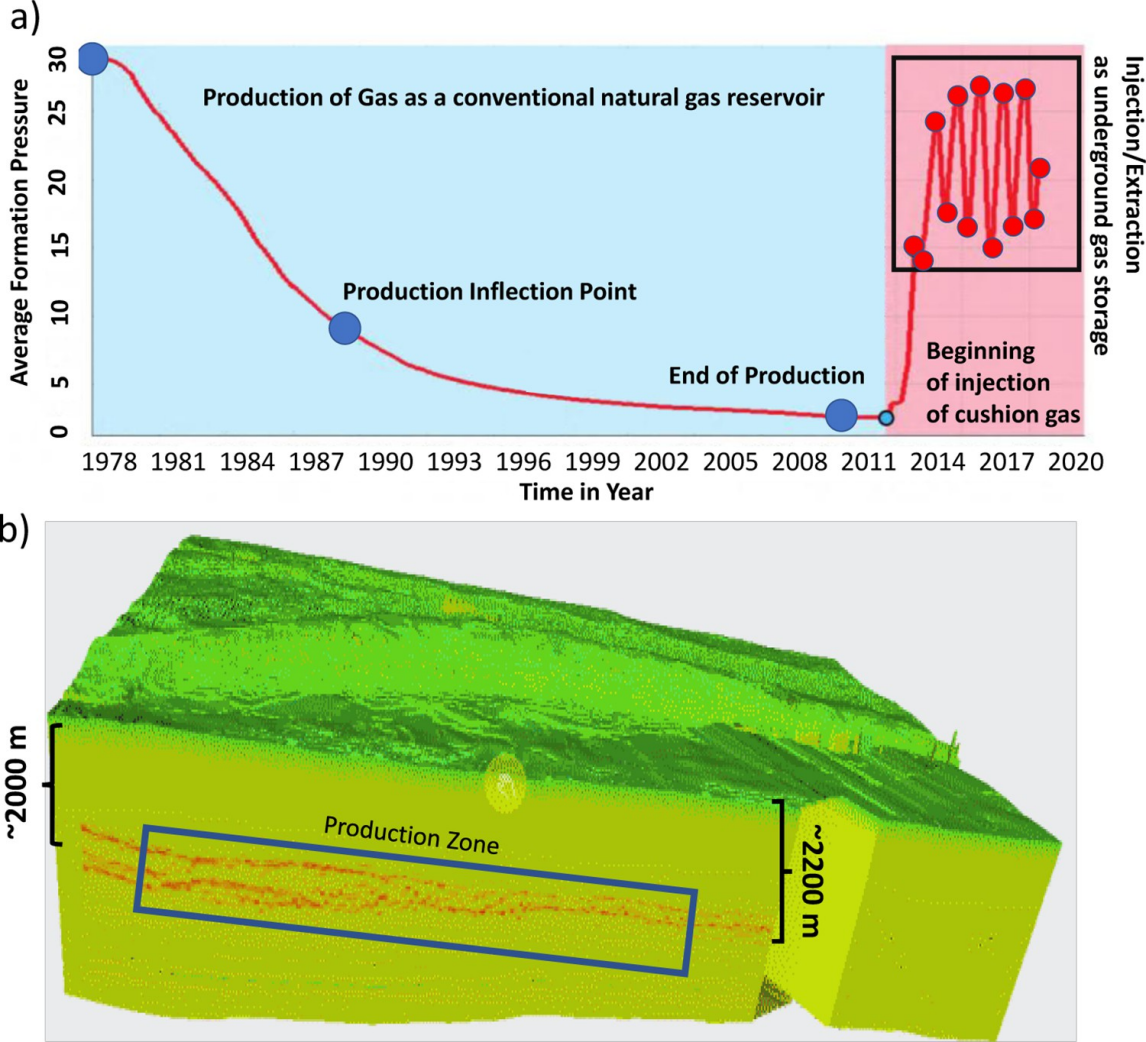
a) the production and later gas storage (injection and production) history of the underground gas storage facility. b) the geological model showing the high permeability zone (gas storage area) and its depth to surface for the XiangGuoSi underground gas storage facility.

One of the initial goals of this mission is to monitor the deformation that might be induced by the gas injection/extraction operations of the XiangGuoSi. Many earlier examples are available that report gas injection/extraction can cause significant surface heave (due to injection) or subsidence (due to extraction) [24, 44, 45]. However, the most directly notable observation in our InSAR images is that it does not show any correlation with the injection/production of gas. The InSAR images are acquired from 2017 to 2023 (see Fig 6). Based on the available records, XiangGuoSi reservoir generally injects gas in the summer times and extracts gas in the winter times–this matches the market demand for natural gas. This annual cycle begin at 2014 and continue into 2020s (Fig 7A)–overlap with our InSAR observation periods.

This observation is surprising, particularly given the seasonal pressure fluctuation of 15MPa (see Fig 7A)–which is rather significant. Nevertheless, the following reasons might explain what we observe.

First, the XiangGuoSi reservoir is deeper than many other natural gas field or underground storage facility. The depth of the XiangGuoSi reservoir is at 2000–2200 meters below the surface. even with the reservoir itself undergoing significant volumetric change, the vertical displacement dissipated when it propagates upward–when the deformation front reaches the surface, the magnitudes had become insignificant.

Second, the most of the geological masses in the areas are withered soft rocks. Most of the geological masses overlaying the XiangGuoSi’s Maokou Formation are the Triassic and Jurassic sediments (see Figs 1B and 7B). These withered sandstones and sandy shale are generally soft and easily compactible. This causes the upward/downward displacement, induced by reservoir expansion/contraction dissipate even faster and further reduces the magnitudes of displacement in the surface.

Last but not the least, or potentially most importantly, the volumetric change of the reservoir is not significant enough. Our records show at least 4 cycles of injection/extraction had been finished during the periods between 2017 and 2021, and the average formation periods remain rather stable. It always peaks at the end of injection at 24-26MPa and reaches the lowest point of 15–18 MPa when the production phase is over. We also observe no long-temporal trends in these pressures, other than the periodical cycles. This suggest reservoir’s rock pore structure is rather stable–even with the pressure variable, the skeleton of the rock still holds on the prevents excessive expansion/contraction. Highly likely, the deformation is entirely elastic and small–resulting even smaller surface deformation that is not detected in our study.

For the long term run of the gas storage, such elasticity and small reservoir deformation is a good sign. Usually, engineers and scientists will worry about the pore spaces being altered, either collapse to cause lower permeability, or induce fracture that causes gas leak. A relatively undeformed and stable pore spaces provide rather constant well injectivity/productivity and long-term stable gas storage capacity.

Further, given the rather linear deformation pattern (see Fig 6), our focus can shift to the areas near the pipeline. Our observed deformation is likely natural and unrelated to the underground gas injection/extraction activity. And, its linearly allows us to use deformation rate and cumulative deformation value as indicators for landslide risk–particularly near the areas of the pipelines. Through this monitoring, ten geological hazard points have been identified. These points have been further classified into three distinct levels based on their severity and potential impact–on the basis of deformation rate, cumulative deformation and proximity to the pipeline (Table 1). These risks are grouped into three different levels of concern, each with different specific response measures.

In Table 1, action 1 stands for increasing the frequency of maintenance personnel inspections; action 2 means regularly using InSAR for targeted monitoring and increase the frequency of maintenance personnel inspections; action 3 is conducting a specialized landslide investigation and use InSAR for continuous long-term monitoring. For category 3, critical attention, if significant instability is consistently observed, additional real-time monitoring methods can be integrated for comprehensive monitoring, and the frequency of inspections should be increased.

Following the risk matrix detailed in Table 1, our points of interests (see Fig 5) and their surrounding areas are categorized into three levels: one area is designated as a key focus area, five areas are classified as continuous focus areas, and four areas are identified as general focus areas.

At the level of key attention, there is a single point of concern 17 meters north of potential pile XH31, located at coordinates 106.686325°E and 29.713503°N. This area is characterized by its proximity to the pipeline combined with high deformation rates or substantial cumulative subsidence. The response for managing this area is robust, involving in-depth investigations and the application of long-term monitoring technologies to mitigate associated risks effectively.

For ongoing attention, five areas have been highlighted due to their varying degrees of subsidence and proximity to the pipeline. These include locations northwest of potential pile TX40, east of potential pile TX64, south of potential pile TX71, around the No. 11 Injection and Extraction Station, and another close to the pipeline. These areas exhibit trends of periodic, continuous, or accelerated subsidence, prompting the need for regular targeted InSAR monitoring alongside increased inspection frequencies by maintenance personnel to manage the evolving geological conditions effectively.

Finally, the areas categorized under general attention also lie near essential infrastructure but are differentiated by lower deformation rates, posing comparatively lower risks. These areas include slopes near potential pile TX7, a depression in farmland northeast of potential pile TX33, a hillside north of Qingyun Reservoir by potential pile TX39, and a steep mountainside beside a road near the No. 5 Injection and Extraction Station. Despite the lower risk, increased inspection frequencies are recommended to ensure continued monitoring and maintenance, keeping the situation under control and preventing any potential escalation in risk levels.

Overall, this tiered monitoring approach allows for tailored management strategies that correspond to the severity and specific characteristics of the geological hazards identified near the gas pipelines, thereby ensuring the safety and integrity of both the infrastructure and the surrounding communities.

## Conclusion

This study employs InSAR, with the data collected between 2017 and 2022, to survey ground deformation in a mountainous region housing an underground natural gas storage facility and the supporting infrastructures–namely the pipeline system. The area under investigation is prone to a range of geohazards, primarily landslides.

Critically, we do not observe correlation between the surface deformation and the underground storage’s cyclic injection/production history. We reasoned that 1. great reservoir depths, 2. soft overlaying rocks and 3. stable reservoir pore spaces, are the causes for this absence of correlation. Particularly, the stable reservoir pore spaces reasoning is supported by the similarly stable formation pressures–that has only cyclic pattern but not long term upward or downward trends. Thus, our observation also suggests the underground gas storage’s long term performance will be stable.

At last, given the linearity of the observed surface deformation, we develop a risk classification guideline that considers total subsidence, deformation rate, and proximity to the pipeline. As such, ten points of interests and the surrounding areas were categorized into three levels: one key focus area, five continuous focus areas, and four general attention areas. The proposed response protocol, based on these risk levels, includes conducting specialized landslide investigations, employing continuous long-term monitoring, and integrating additional real-time monitoring methods if significant instability is consistently detected.

## Supporting information

S1 File InSAR data used for this work. (7Z)

S1 Appendix (DOCX)
