# Supplementary material for: Investigating geohazard risk in mountainous areas for underground gas storage using InSAR and development of a protocol for hazard prevention
Source: PLoS One. 2025 Feb 13;20(2):e0318860. doi: 10.1371/journal.pone.0318860 (PMC11825013; doi:10.1371/journal.pone.0318860)
Supplement: S1 Appendix — (DOCX) [file pone.0318860.s002.docx]

# Appendix:

**Table A1. InSAR scene data used for this work.**

| **Scene** | **Date** | **Interval**  **(days)** | **Scene** | **Date** | **Interval**  **(days)** | **Scene** | **Date** | **Interval**  **(days)** |
| --- | --- | --- | --- | --- | --- | --- | --- | --- |
| 1 | 20170327 | 12 | 54 | 20190128 | 12 | 107 | 20201025 | 12 |
| 2 | 20170408 | 12 | 55 | 20190209 | 12 | 108 | 20201106 | 12 |
| 3 | 20170420 | 12 | 56 | 20190221 | 12 | 109 | 20201118 | 12 |
| 4 | 20170502 | 12 | 57 | 20190305 | 12 | 110 | 20201130 | 12 |
| 5 | 20170514 | 12 | 58 | 20190317 | 12 | 111 | 20201212 | 12 |
| 6 | 20170526 | 12 | 59 | 20190329 | 12 | 112 | 20201224 | 12 |
| 7 | 20170607 | 12 | 60 | 20190410 | 12 | 113 | 20210105 | 12 |
| 8 | 20170619 | 12 | 61 | 20190422 | 12 | 114 | 20210117 | 12 |
| 9 | 20170701 | 12 | 62 | 20190504 | 12 | 115 | 20210129 | 12 |
| 10 | 20170713 | 12 | 63 | 20190516 | 12 | 116 | 20210210 | 12 |
| 11 | 20170725 | 12 | 64 | 20190528 | 12 | 117 | 20210222 | 12 |
| 12 | 20170806 | 12 | 65 | 20190609 | 12 | 118 | 20210306 | 12 |
| 13 | 20170818 | 12 | 66 | 20190621 | 12 | 119 | 20210318 | 12 |
| 14 | 20170830 | 12 | 67 | 20190703 | 12 | 120 | 20210330 | 12 |
| 15 | 20170911 | 12 | 68 | 20190715 | 12 | 121 | 20210411 | 12 |
| 16 | 20170923 | 12 | 69 | 20190727 | 12 | 122 | 20210423 | 12 |
| 17 | 20171005 | 12 | 70 | 20190808 | 12 | 123 | 20210529 | 36 |
| 18 | 20171029 | 24 | 71 | 20190820 | 12 | 124 | 20210610 | 12 |
| 19 | 20171110 | 12 | 72 | 20190901 | 12 | 125 | 20210622 | 12 |
| 20 | 20171122 | 12 | 73 | 20190913 | 12 | 126 | 20210716 | 24 |
| 21 | 20171204 | 12 | 74 | 20190925 | 12 | 127 | 20210728 | 12 |
| 22 | 20171216 | 12 | 75 | 20191007 | 12 | 128 | 20210809 | 12 |
| 23 | 20171228 | 12 | 76 | 20191019 | 12 | 129 | 20210821 | 12 |
| 24 | 20180109 | 12 | 77 | 20191031 | 12 | 130 | 20210902 | 12 |
| 25 | 20180121 | 12 | 78 | 20191112 | 12 | 131 | 20210914 | 12 |
| 26 | 20180202 | 12 | 79 | 20191124 | 12 | 132 | 20210926 | 12 |
| 27 | 20180214 | 12 | 80 | 20191206 | 12 | 133 | 20211008 | 12 |
| 28 | 20180226 | 12 | 81 | 20191218 | 12 | 134 | 20211020 | 12 |
| 29 | 20180310 | 12 | 82 | 20191230 | 12 | 135 | 20211101 | 12 |
| 30 | 20180322 | 12 | 83 | 20200111 | 12 | 136 | 20211113 | 12 |
| 31 | 20180403 | 12 | 84 | 20200123 | 12 | 137 | 20211125 | 12 |
| 32 | 20180415 | 12 | 85 | 20200204 | 12 | 138 | 20211207 | 12 |
| 33 | 20180427 | 12 | 86 | 20200216 | 12 | 139 | 20211219 | 12 |
| 34 | 20180509 | 12 | 87 | 20200228 | 12 | 140 | 20211231 | 12 |
| 35 | 20180521 | 12 | 88 | 20200311 | 12 | 141 | 20220112 | 12 |
| 36 | 20180602 | 12 | 89 | 20200323 | 12 | 142 | 20220124 | 12 |
| 37 | 20180614 | 12 | 90 | 20200404 | 12 | 143 | 20220205 | 12 |
| 38 | 20180626 | 12 | 91 | 20200416 | 12 | 144 | 20220217 | 12 |
| 39 | 20180708 | 12 | 92 | 20200428 | 12 | 145 | 20220313 | 24 |
| 40 | 20180720 | 12 | 93 | 20200510 | 12 | 146 | 20220406 | 24 |
| 41 | 20180801 | 12 | 94 | 20200522 | 12 | 147 | 20220418 | 12 |
| 42 | 20180825 | 24 | 95 | 20200603 | 12 | 148 | 20220430 | 12 |
| 43 | 20180906 | 12 | 96 | 20200615 | 12 | 149 | 20220524 | 24 |
| 44 | 20180918 | 12 | 97 | 20200627 | 12 | 150 | 20220629 | 36 |
| 45 | 20180930 | 12 | 98 | 20200709 | 12 | 151 | 20220711 | 12 |
| 46 | 20181012 | 12 | 99 | 20200721 | 12 | 152 | 20220723 | 12 |
| 47 | 20181024 | 12 | 100 | 20200802 | 12 | 153 | 20220804 | 12 |
| 48 | 20181105 | 12 | 101 | 20200814 | 12 | 154 | 20220828 | 24 |
| 49 | 20181129 | 24 | 102 | 20200826 | 12 | 155 | 20220909 | 12 |
| 50 | 20181211 | 12 | 103 | 20200907 | 12 | 156 | 20220921 | 12 |
| 51 | 20181223 | 12 | 104 | 20200919 | 12 | 157 | 20221120 | 60 |
| 52 | 20190104 | 12 | 105 | 20201001 | 12 | 158 |  |  |
| 53 | 20190116 | 12 | 106 | 20201013 | 12 | 159 |  |  |
